# Population Structure of *Phytophthora infestans* in Israel Changes Frequently Due to the Import of Asymptomatic Late Blight-Infected Potato Seed Tubers from Europe

**DOI:** 10.3390/jof10080549

**Published:** 2024-08-04

**Authors:** Yigal Cohen

**Affiliations:** Faculty of Life Sciences, Bar Ilan University, Ramat Gan 5290002, Israel; ycohen@biu.ac.il or yigal.cohen1@gmail.com

**Keywords:** CAA fungicides, disease control, EuroBlight, OSBPI fungicides, mandipropamid, oomycetes, oxathiapiprolin, tomato

## Abstract

Late blight, caused by the oomycete *Phytophthora infestans*, is a devastating disease of potato worldwide. In Israel, potatoes are grown twice a year, in autumn and spring, with late blight causing extensive damage in both seasons. While tuber seeds for the autumn planting are produced locally, seed tubers for the spring planting are imported from Europe due to dormancy of local tubers. Here, we demonstrate that seed tubers imported from Europe for the spring season carry asymptomatic infection with EU genotypes of *P. infestans*, which alters the population structure of the pathogen each spring. The proportion of imported tubers carrying asymptomatic infections ranged between 1.2 and 3.75%, varying by year and cultivar. Asymptomatic tubers produced late blight-infected sprouts about one month after planting. The sporangia produced on these sprouts served as primary inoculum, causing intensive foliage attacks on neighboring plants. When sprout-infected plants were uprooted and the mother tuber was washed, sliced, and placed in moistened dishes at 18 °C, profuse sporulation of *P. infestans* developed on the slices’ surfaces within 1–2 days. The dominant genotype of *P. infestans* in the autumn season in Israel is 23A1, but genotypes in the following spring season changed to include 13A2 or 36A2. Surprisingly, genotype 43A1, which might be resistant to CAA and OSBPI fungicides and appeared in Europe in 2022, emerged in Israel in spring 2024. The immigrating genotypes do not persist in the country, allowing 23A1 to regain predominance in the following autumn. Long-term monitoring data suggest that the population structure of *P. infestans* changes yearly but temporarily due to the import of new genotypes from Europe.

## 1. Introduction

Potatoes are grown in Israel twice a year, in autumn (planting in September–October, harvest in December–January) and spring (planting in December–January, harvest in April–May). Tuber seeds for the autumn season are produced locally in the previous spring season, while tuber seeds for the spring planting are imported yearly from Europe due to the dormancy of local tubers. Late blight, caused by *Phytophthora infestans* (Mont.) DeBary, is a devastating disease in both seasons, attacking from November until May.

The population structure of *P. infestans* in Israel changes frequently, mainly in the spring season. The reasons responsible for these changes are poorly understood. The objective of this study was to provide adequate explanations for why and how these changes in the population structure take place.

Monitoring the population structure of *P. infestans* in Israel began in 1983 when A2 mating type metalaxyl-resistant (R) isolates emigrated from Europe to the country [1]. Between 1983 and 1991, clonal populations of exclusively A2 mating types were found, with a steady increase in the frequency of R isolates over the years. From 1993 until 2012, the A1 mating type dominated and coexisted with the A2 mating type, suggesting that sexual recombination most likely occurred [2,3], allowing for isolates partially resistant to metalaxyl (intermediate, I) to appear. The frequency of R isolates drastically declined from 1993 to 2012 [4]. Thus, *P. infestans* underwent three major genetic changes during the period 1983–2000 [5]. The A2 R isolates were more competitive than A1 S (metalaxyl-sensitive) isolates on potato foliage [6], whereas the A1 S isolates were more competitive in tubers under storage [7,8].

From 2004 onward, monitoring of the potato population of *P. infestans* in Israel was accompanied by SSR genotype analysis. The present study describes the population structure of *P. infestans* in Israel over the last 21 years (2004–2024). It shows that the population structure significantly changed each spring but did not persist into the next autumn season. This study also proves that imported seed tubers from the EU are responsible for the changes in the spring. The entrance of new genotypes into the country in the spring occurred via asymptomatic seed tubers carrying latent infections of *P. infestans*.

## 2. Materials and Methods

### 2.1. Latent Infection in Imported Tubers—2016

Certified potato seed tubers cv Nicola were imported from Holland (producer unknown; supplied by Yaham Ltd., Magen, Israel). Five hundred tubers were first inspected visually for the absence of late blight symptoms and thereafter sown in soil on 6 December 2016 inside a low net house (No 1, 7 × 3 × 100 m) covered with 50 mesh white screen, located on campus. The soil had not been exposed to potato or tomato cultivation in the past 10 years. The plants were drip-irrigated twice a week with water containing 0.5% NPK fertilizer.

### 2.2. Latent Infection in Imported Tubers—2017

Two hundred seed tubers of cv Nicola and 200 seed tubers of cv Mondial (both imported from Europe (unknown producer; supplied by Yaham Ltd., Magen, Israel) were first inspected visually, to verify that there were no late blight symptoms, and thereafter sown on 5 January 2017 in soil in net house No 9 on campus. The soil had not been exposed to potato or tomato cultivation in the past 10 years. Another 200 seed tubers of cv Mondial were sown after similar inspection in a high net house (No 1, 16 × 7 × 25 m) in 120 L (0.5 × 0.2 × 1.2 m) polystyrene containers filled with a pasteurized peat:perlite (10:1) mixture, with 5 tubers per container. The plants were drip-irrigated twice a week with water containing 0.5% NPK fertilizer.

### 2.3. Latent Infection in Imported Tubers—2024

Certified potato seed tubers of 8 cultivars were imported from Europe (supplied by Yaham Ltd., Magen, Israel). Table 1 provides the names of the cultivars and their European producers. Eighty tubers of each cultivar were first inspected visually, to verify that there were no late blight symptoms, and thereafter sown on 1 January 2024 in 120 L (0.5 × 0.2 × 1.2 m) polystyrene containers filled with a pasteurized peat:perlite (10:1) mixture, with 5 tubers per container. The containers were placed inside a high net house (No 7, 7 × 5 × 50 m) located on campus. The plants were drip-irrigated twice a week with water containing 0.5% NPK fertilizer. Weather data were retrieved from a local meteorological station.

### 2.4. Collection of Field Isolates 2004–2024

About 1120 isolates of *P. infestans* were collected from infected potato fields in the western Negev, Israel, during the autumn and spring seasons of 2004–2024 (30–40 isolates per season). Samples of 5–10 infected leaves were collected from an infected field, placed in a moistened plastic bag in a cooler, and shipped to the laboratory within a few hours. Infected leaves were placed on moist filter paper in 14 cm Petri dishes and incubated at 18 °C in the dark for ~15 h to allow for sporulation of the pathogen. The produced sporangia were harvested into ice-cold distilled water and used for propagation and DNA extraction. To propagate an isolate, sporangia were drop-inoculated onto detached leaflets of potato (Sifra) or tomato (Roter Gnom) and placed for a week in a growth chamber at 18 °C for sporulation as described before [9].

### 2.5. Extraction of DNA from Sporangia of Phytophthora Infestans

DNA was extracted as described for *Pseudoperonospora cubensis* [10]. Briefly, a sample of about 1 × 10^6^ sporangia was macerated in 1.5 mL micro-tubes using disposable pellet pestle grinders. Maceration continued after adding 0.6 mL CTAB (hexadecyltrimethyl-ammonium bromide) buffer [1.4 M NaCl, 20 mM EDTA, 100 mM TRIS-Cl, 2% (*w*/*v*) CTAB pH 8.0], and the sample was incubated at 60 °C for 45 min. The sample was then extracted with 0.6 mL chloroform/isoamyl alcohol (24:1) and centrifuged at 12,000× *g* for 5 min. The aqueous phase was transferred to a 1.5 mL tube where the DNA was precipitated with an equal volume of cold (−20 °C) isopropanol. DNA concentration was determined with a ND-1000 spectrophotometer (NanoDrop, Waltham, MA, USA).

### 2.6. Genotype Identification

The DNA samples were shipped to Dr. David Cooke (The James Hutton Institute, Invergowrie, Dundee DD2 5DA, UK). Genotyping of the samples and reference isolates was performed as described by Li et al. [11] using a 12-plex PCR technique employing multiplexing of twelve SSR markers. The markers used in this study were D13, G11, Pi4B, Pi04, Pi63, Pi70, PinfSSR2, PinfSSR3, PinfSSR4, PinfSSR6, PinfSSR8, and PinfSSR11 [12].

### 2.7. Data Analysis

The percentage of leaf area infected with late blight in the 2024 experiment was visually assessed in each of the 8 cultivars from 41 days after planting until 76 days after planting. Tukey’s HSD (honestly significant difference) test was performed to detect significant differences at =0.05 level between the mean late blight-infected leaf area of the 8 potato cultivars at 76 days after planting.

## 3. Results

### 3.1. Latent Infection 2016

**Figure 1:**
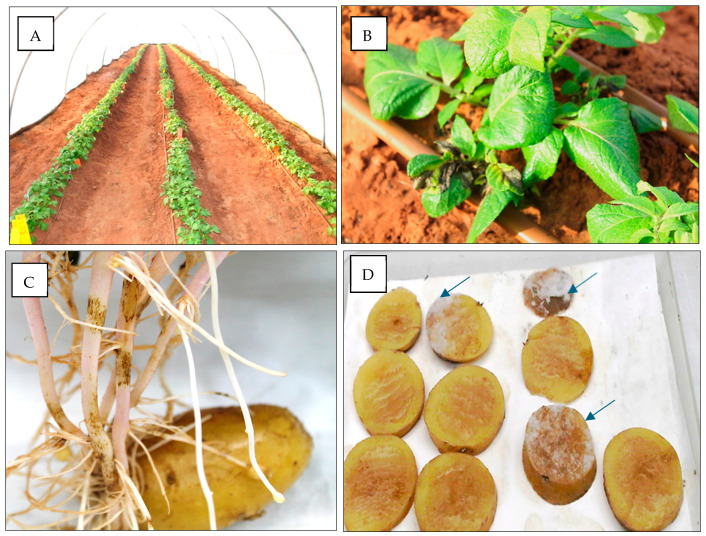
Potato seed tubers (*cv* Nicola, imported from Holland) carrying asymptomatic infection with *Phytophthora infestans* developed late blight symptoms upon germination. Tubers were sown on 6 December 2016. Symptoms were seen on 11 January 2017, 36 days after planting. (**A**) The appearance of 500 plants 5 weeks after sowing. (**B**) Emerging sprouts showing late blight symptoms. (**C**) Below-ground stems showing necrotic symptoms of late blight, while mother tuber appears healthy. (**D**) Sporulation of *Phytophthora infestans* on tuber slices (arrows) that were taken from the mother tuber shown in (**C**).

Imported tubers were sown in soil on 6 December 2016. On 11 January 2017 (36 days after planting), 6 out of 500 (1.2%) emerging plants showed late blight symptoms on sprouts. The infected plants were scattered randomly in the net house (Figure 1A). Symptoms were visible on the leaves and/or stem (Figure 1B). No late blight symptoms were visible on the skin of the mother seed tubers when the symptomatic plants were uprooted from the soil, whereas symptoms were seen on the below-ground emerging stems (Figure 1C). The six infected plants were uprooted, their mother tubers were washed, surface sterilized, sliced, and the slices placed on a dry filter paper in dishes at 18 °C in the dark for 48 h. Profuse sporulation of *P. infestans* was observed on the slices of all mother tubers (Figure 1D). No such sporulation occurred in mother tubers taken from neighboring healthy plants (n = 10). SSR analysis of sporangia collected from the sporulating slices revealed genotype 13A2. Virulence analysis revealed a complex race 123456791011 of *P. infestans*, intermediately resistant to metalaxyl. The largest number of isolates belonging to genotype 13A2 were collected during spring 2016 (see below).

### 3.2. Latent Infection 2017

**Figure 2:**
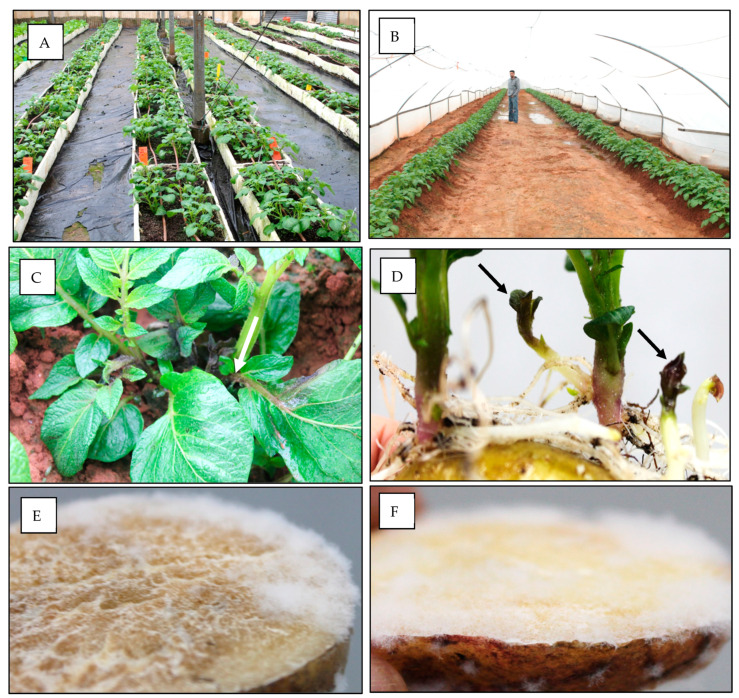
Potato seed tubers imported from Europe carrying asymptomatic infection with *Phytophthora infestans* developed late blight symptoms upon germination. Tubers were sown on 5 January 2017. Symptoms were detected on 15 February 2017, 41 days after sowing. (**A**) Nicola plants in net house 1. (**B**) Mondial plants in net house 9. (**C**) symptoms (arrow) of late blight at ground level. (**D**) symptoms of late blight on sprout apex (arrows). (**E**) sporulation of *Phytophthora infestans* on a tuber slice of cv Nicola. (**F**) Sporulation of *Phytophthora infestans* on a tuber slice of cv Mondial.

The imported tubers were sown on 5 January 2017. On 15 February 2017 (41 days after planting), 4 out 400 (1%) Nicola plants and 1 out of 200 (0.5%) Mondial plants showed late blight symptoms on sprouts (Figure 2A–D). Slices taken from the mother tubers of infected plants showed heavy sporulation of *P. infestans* (Figure 2E,F).

### 3.3. Latent Infection 2024

**Figure 3:**
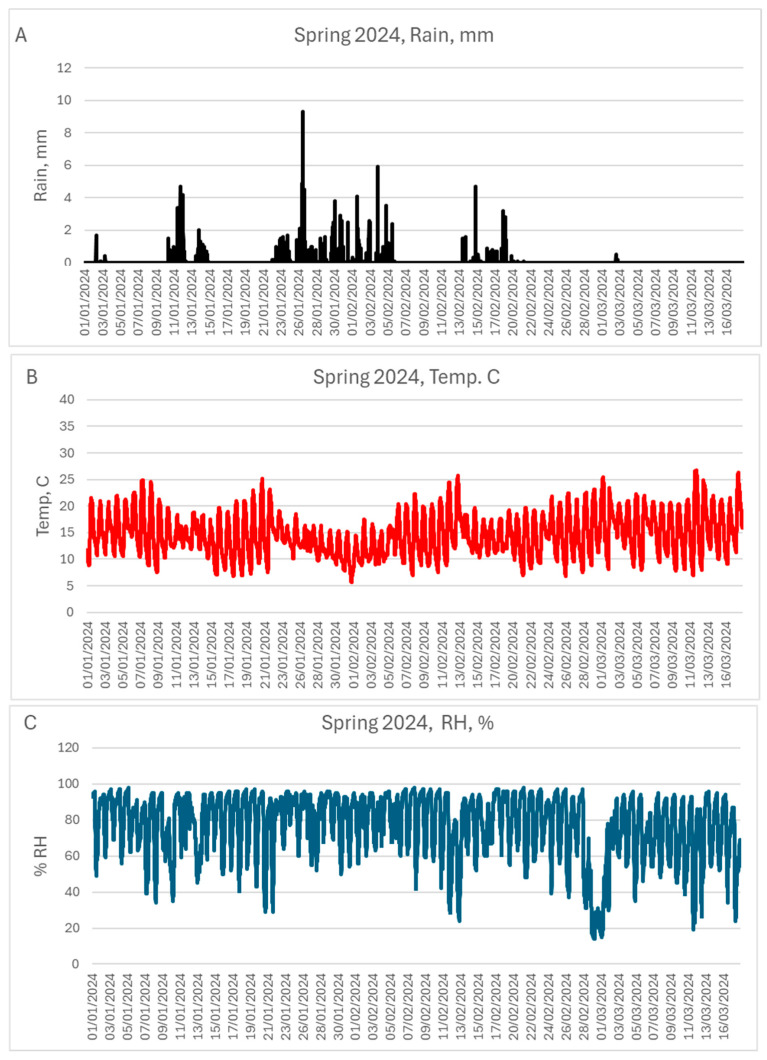
Meteorological conditions prevailing in Spring 2024 at BIU Farm during the epidemics of late blight caused by *Phytophthora infestans* in eight cultivars of potato whose seeds were imported from Europe. (**A**) rain (total = 370 mm). (**B**) air temperature (mean = 14.6 °C; min = 5.8 °C; max = 26.6 °C). (**C**) % RH (mean = 76.2%; min = 14%; max = 98%).

**Figure 4:**
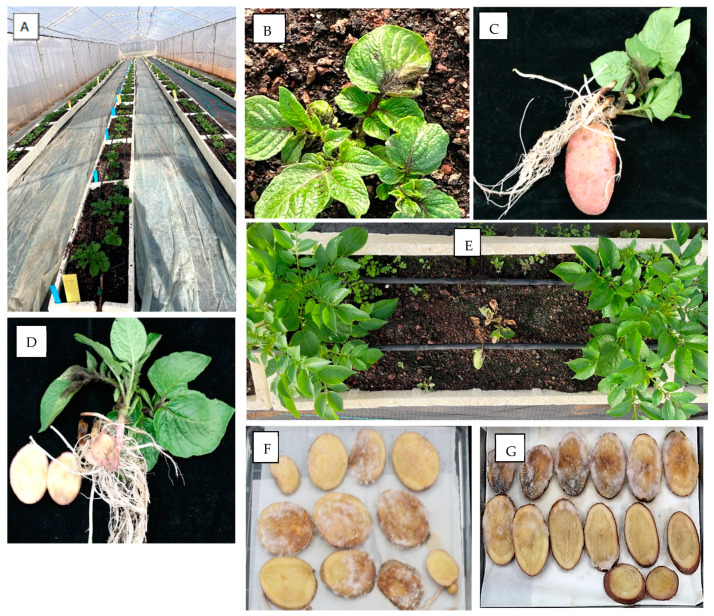
Potato seed tubers (*cv* Rosana and VR 808) carrying asymptomatic infection with *Phytophthora infestans* developed late blight symptoms upon germination. Imported tubers were sown on 1 January 2024 and sprout symptoms were observed on 7 February 2024, 36 days after sowing. (**A**) Net house with germinating potato plants at 36 days after planting. (**B**–**D**) Late blight symptoms on a germinating plant cultivar Rosana with no external symptoms on tubers. (**E**) An infected plant of cv VR 808 at 40 days after planting. Note the two healthy plants alongside. (**F**,**G**) Sporulation of *P. infestans* on surface of tuber slices cut from symptomless tubers of VR-808 and Rosana, respectively.

Eighty seed tubers of each of the eight cultivars (Table 1) were sown on 1 January 2024 in 128 containers filled with pasteurized soil in a net house at BIU Farm. The meteorological conditions during the growing season were conducive to late blight development (Figure 3). On 7 February 2024, 37 days after sowing, infected sprouts were visible in two Rosana plants and three VR-808 plants (Figure 4A–F). Upon uprooting the plants from the soil and washing, the mother tubers showed no symptoms of late blight. However, when the tubers were sliced and placed in moistened dishes, profuse sporulation of *P. infestans* was observed on the slices’ surfaces (Figure 4F,G).

**Figure 5:**
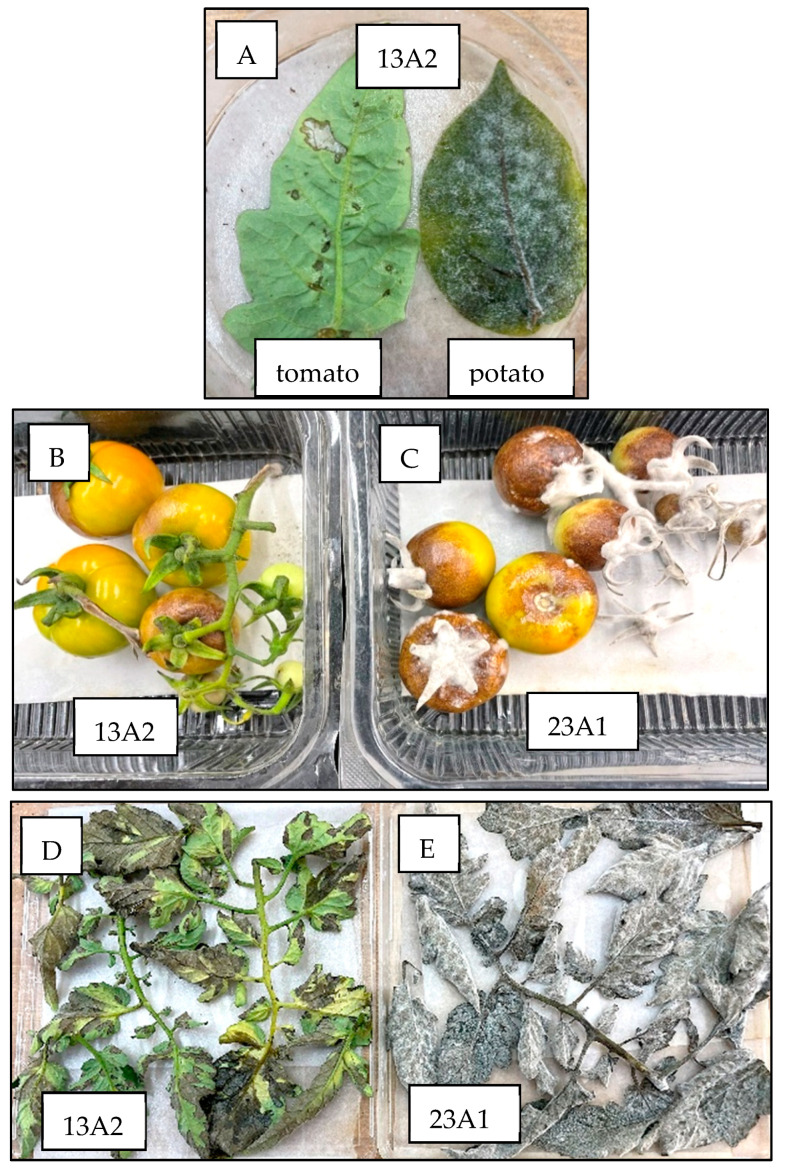
Compatibility to potato and tomato of genotypes 23A1 and 13A2 retrieved from potato. (**A**) In detached tomato and potato leaves. (**B**,**C**) In tomato fruits. (**D**,**E**) In tomato leaves. Note heavy sporulation of 23A1 on tomato fruits and leaves as against hypersensitive response to 13A2 with no sporulation.

On 15 February 2024, 45 days after planting, the disease appeared across the net house in all cultivars, making it impossible to determine whether the source of infection was aerial or from tubers. Leaf samples were collected from each cultivar separately, and the sporangia produced thereafter in moistened Petri dishes were subjected to several tests and SSR analysis. A similar collection of infected leaves was performed at 76 days after planting. The traits of sporangia collected from the tubers slices and leaves are shown in Table 2. The isolates retrieved from the seed tubers belonged to the 13A2 genotype. The isolates collected from the leaves at 45 days after planting belonged to genotypes 23A1, 13A2 or 43A1, depending on the cultivar, whereas those collected at 76 days after planting all belonged to 13A2, confirming the highly competitive virulence of this genotype. The retrieved 13A2 isolates were all resistant to mefenoxam and infective to 10 out 11 potato differential lines (race 12345678910). They were all incompatible with tomatoes (Figure 5). When inoculated with a low sporangial dose (10 sporangia/droplet), they sporulated on potato leaves but produced no symptoms on tomato leaves (Figure 5A). When isolates 23A1 or 13 A2 were spray-inoculated at a high sporangial dose (5000 sporangia/mL), the former produced heavy sporulation on fruits (Figure 5B) and leaves (Figure 5C) of tomato, whereas the latter produced symptoms with no sporulation on fruits or leaves of tomato.

**Figure 6:**
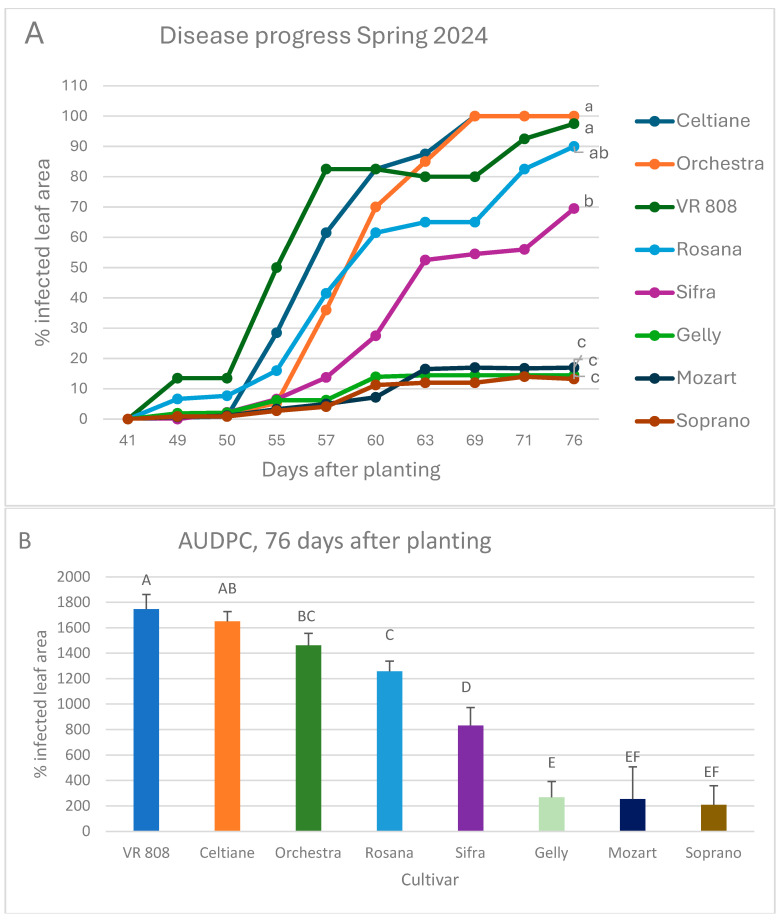
Progress of late blight on foliage of eight cultivars of imported potato cultivars. (**A**) Disease progresses in each cultivar during a 76-day period after planting. (**B**) Area under disease progress curves (derived from data in (**A**)). Different letters on curves or columns indicate a significant difference between cultivars at α = 0.05 (Tukey’s HDS).

Large differences were observed in the progress of late blight on the foliage of the eight potato cultivars in the net house (Figure 6A). The most susceptible cultivars were VR 808, Rosana and Celtiane, while the least susceptible ones were Gelly, Mozart, and Soprano (Figure 6B).

### 3.4. Population Structure 2004–2024

**Figure 7:**
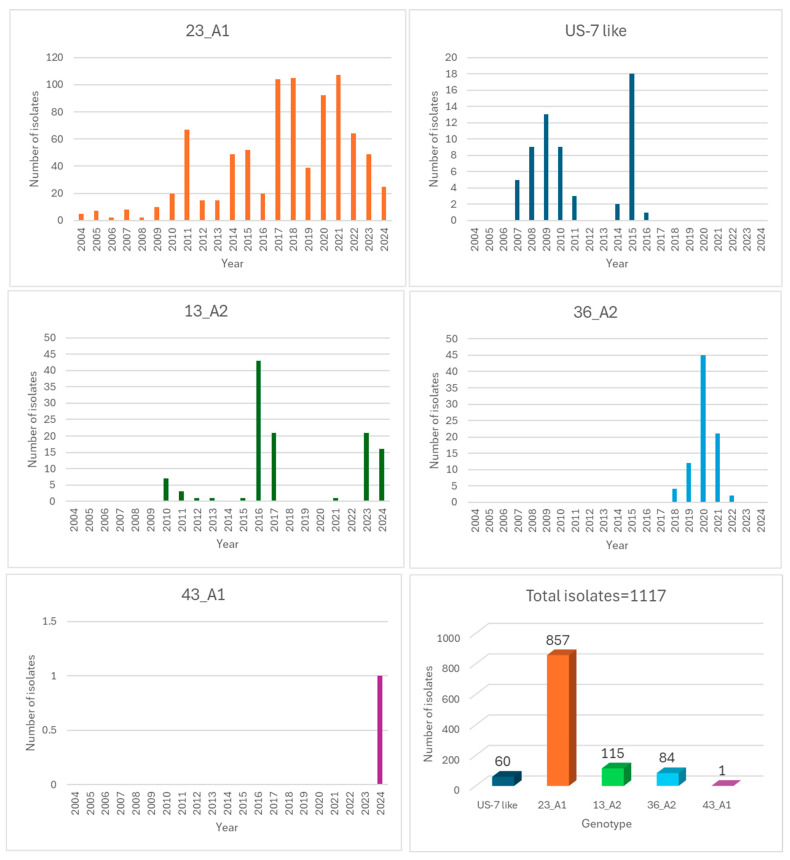
Annual frequency of genotypes of *Phytophthora infestans* in potato crops in Israel during the period 2004–2024.

Five genotypes of *P. infestans* were detected in potato fields in Israel during the past 21 years: 23A1, US7-like, 13A2, 36A2, and 43A1. The yearly fluctuation in their frequency is shown in Figure 7. The total number of isolates belonging to these genotypes was 857, 60, 115, 84, and 1, respectively.

Genotype 23A1 appeared in 2004, US7-like in 2007, 13A2 in 2010, 36A2 in 2018, and 43A1 appeared in spring 2024. They were detected in the country during 21, 8, 10, 5, and 1 year(s), respectively. Their order of appearance and decline in Israel may reflect the order of their appearance and decline in Europe (EuroBlight) (See Table 3).

Table 3 shows the seasonal shift (autumn vs. spring) in the population structure during the last 17 years (2007–2024). (No such data are available for 2004–2006.) The data confirm that the detection of genotypes 13A2 and 36A2 mostly occurred in the spring season, probably harbored inside infected seed tubers imported from Europe. Thus, out of 116 isolates that belonged to genotype 13A2, 108 (93.1%) were detected in the spring seasons. Similarly, out of 84 isolates that belonged to genotype 36A2, 74 isolates (88.1%) were detected in the spring. Genotype 43A1 showed up in spring 2024, soon after its dramatic appearance in Europe with resistance to CAA and OSBPI fungicides. Out of 60 isolates that belonged to US7-like, 36 isolates (60%) appeared in the spring. In contrast, genotype 23A1 was more frequent in the autumn season (55% of the isolates) as compared to the spring season (45% of the isolates), suggesting a local over-summering.

## 4. Discussion

Global trade of potato tubers facilitates the worldwide transportation of *Phytophthora infestans*. This has been occurring since the 1840s when *P. infestans* first migrated northward from Peru to Colombia, Mexico, the USA, and then to Europe and Asia [13]. From Europe, the pathogen has spread to other countries, including India [14,15], Egypt [12], north Africa, and South Africa [16]. Detailed routes of migration in Europe, the USA, Asia, and Latin America are available from the internet sites EuroBlight, USABlight, AsiaBlight, and Tizón Latino, respectively.

Reports in EuroBlight confirm that all five genotypes that were detected in Israel were previously detected in Europe. Genotype 23A1 has been prevalent since 2004 on tomatoes in south Europe. In 2023, it comprised most of the population in Italy. Genotype 13A2 was first detected in Europe, in Holland, and Germany, in 2004. It reached Britain in 2005 and displaced the population by 2006 [17]. It dominated the population for a decade and then declined to 9.3% in 2019, 7.6% in 2020 and 4.9% in 2021. Genotype 36A2 appeared in Europe in 2015. It rose from 20.8% in 2020 to 36.7% in 2021. In 2023, it comprised 46, 64 and 76% of the samples in Belgium, England, and France, respectively. Comprising 37% of the 2023 samples, genotype 36A2 was the most frequently sampled genotype, suggesting it remains fitter than other clones. The frequency of genotype 43A1 increased from 17% in 2022 to 23% of the population in 2023. It has extended its range to ten European countries [18,19].

In this paper, we show that *P. infestans* is continuously migrating from Europe to Israel via potato tuber seeds. These migrations occur through asymptomatic infected tubers, which are undetectable by buyers during field inspections before harvest and by growers at the time of sowing. Asymptomatic infected tubers only manifest the disease on germinating sprouts about one month after sowing in soil.

The asymptomatic infection of potato tubers with *P. infestans* remains a biological mystery. It is unclear how and when the pathogen reaches the tubers, whether mycelia/sporangia occupy the tuber buds (eyes) or lenticels, or whether the mycelium has penetrated the parenchyma. Additionally, it is unknown why external rot does not develop, nor is it known what the mechanism that keeps the pathogen silent during tuber dormancy until sprouting. This behavior might mimic *Uncinula necator*, the powdery mildew fungal agent of grapevine, which overwinters in buds of the twigs and develops heavily mildewed “flag” shoots in the spring, thus serving as a source of primary inoculum [20].

Using artificial inoculation of tuber buds, Johnson and Cummings [21] observed that latent infection of potato seed tubers and the production of viable sporangia of *P. infestans* occurred after long-term cold storage at around 4 °C. Latent infection in tubers was asymptomatic, without discoloration or necrosis in external or internal tissues at the end of storage. However, symptoms and sporangia developed on asymptomatic tubers placed at temperatures of 15 °C and above. They concluded that *P. infestans* could survive asymptomatically in potato seed tubers for extended periods at around 4 °C. Latent infection of seed tubers poses a challenge for late blight management as visual inspection will not reveal latently infected tubers, and tubers with low infection severity may be overlooked. In another report, Johnson [22] showed that mist periods favored the expression of *P. infestans* in infected shoots emerging from late blight-infected seed tubers. Moist conditions that favor the emergence of infected shoots also favor sporulation and repeated infections in the field.

Unlike these reports, our potato seed tubers showed no symptoms after three weeks of incubation at 20–25 °C. In our 2016, 2017 and 2024 trials, a small proportion (1–3.75%) of asymptomatic seed tubers developed infected plants in the field. Heavy rains were associated with the emergence of infected sprouts from the asymptomatic late blight-infected seed tubers. Of the eight cultivars tested in 2024, Rosana and VR 808 showed the emergence of infected sprouts. In all trials, mother tubers of the infected plants produced heavy sporulation of *P. infestans* upon slicing. SSR analysis revealed the arrival of genotypes 13A2 and 43A1 from Europe. This is the first report on the occurrence of 43A1 in Israel.

The genotype 23A1 has predominated in the Israeli population for the past 21 years, comprising about 76.7% of all isolates. It has replaced genotype US7-like, and its long persistence is likely related to its compatibility with tomatoes, which are abundantly grown during the summer (June–November) in areas adjacent to potato-growing regions in the country. No experimental data are available to prove its competitive fitness on potato with other genotypes of *P. infestans* present in the Israeli population. This genotype is also common in north Africa and South Africa [16]. In Egypt, SSR genotyping of 152 isolates collected from potato and tomato (and one from eggplant) during 2013–2018 showed that, in contrast to 2010–2012 [23], when the proportion of the 13A2 lineage was 35%, all later isolates belonged to the 23A1 lineage [12]. The widespread distribution of the 23A1 clonal lineage on both potato and tomato crops in Egypt could be attributed to its adaptation to Egyptian climatic conditions [12]. Some isolates of the 23A1 lineage might have become tolerant to warmer Egyptian agro-ecological conditions [23].

Genotype 43A1 arrived in Israel in spring 2024 in infected asymptomatic seed tubers produced in Europe in summer 2023. This genotype has recently increased in the EU population. Recent reports by EuroBlight indicated a transition to relatively new genotypes such as EU36, EU37, EU41, EU43, and EU46, with 68% of the samples being genotypes unknown nine years ago. Conversely, the older genotypes, such as EU8A1, EU6A1, and EU13A2, only represented 17% of the population compared to 68% in 2014. Genotype EU43 increased from 2% of the sampled European population in 2021 to 17% in 2022, and to 23% in 2023. Marked increases in the frequency of EU43 were observed in The Netherlands (from 42% to 55%), Germany (from 7% to 50%), and Belgium (from 12% to 35%) from 2022 to 2023. The sampled range of EU43 also widened to include France, Ireland, and Spain in 2023, in addition to the seven other countries where it was previously reported. EU43 has been reported to be resistant to the CAA and OSBPI groups of fungicides [18,19,24,25]. Industry reports show that there are resistant and sensitive isolates to both fungicide groups in genotype EU43 [19]. It is highly likely that the reported resistances account for the dominance of the EU43 lineage in the intensively sampled regions of The Netherlands, Belgium, and northern Germany in 2023. According to EuroBlight, these later changes in *P. infestans* populations may directly influence the efficacy of plant protection products, the deployment of resistant cultivars, and the performance of disease warning systems.

Our unpublished data suggest that the EU43A1 isolate that has arrived in Israel in spring 2024 is sensitive to 0.3 ppm ai oxathiapiprolin.

In conclusion, the potato industry in Israel is continuously threatened with new late blight genotypes immigrating to the country from Europe via asymptomatic seed tubers. Disease management should include strict visual inspection of the newly sown potato fields in the spring season as soon as sprouts emerge from soil.

## Figures and Tables

**Table 1:** List of the potato cultivars used in this study, including their origin and producer.

Cultivar	Producer	Country of Origin
Celtiane	Germicopa	France
Gelly	Eroplant	Germany
Mozart	HZPC	Holland
Orchestra	Meijer Potato	Holland
Rosana	Germicopa	Holland
Sifra	HZPC	Holland/France
Soprano	Meijer Potato	Holland
VR 808	Saltire Seeds	Scotland

**Table 2:** Genotypes of *Phytophthora infestans* retrieved from seed tubers (at 38 days after planting) or leaves (at 45 and 88 days after planting) of seven cultivars of potato. Seed tubers were imported from Europe (see Table 1) and sown on 1 January 2024 in pasteurized soil in a net house at BIU Farm. (No data are available for cultivar Celtiane). nt = not tested. Each genotype is marked with a specific background color.

		Genotype	
	38 Days	45 Days	88 Days
Cultivar	Seed Tubers	Leaves	Leaves
VR 808	EU_13_A2	EU_23_A1	EU_13_A2
Rosana	EU_13_A2	EU_13_A2	EU_13_A2
Gelly	nt	EU_13_A2	EU_13_A2
Orchestra	nt	EU_13_A2	EU_13_A2
Soprano	nt	EU_43_A1	EU_13_A2
Mozart	nt	EU_13_A2	EU_13_A2
Sifra	nt	EU_23_A1	EU_13_A2

**Table 3:** Population structure of *Phytophthora infestans* in potato crops in Israel for the 17-year period of 2007–2024. Figures with a background color represent the number of isolates that were collected in the spring season. Each genotype has a different background color.

		Genotype and Number of Isolates				
Year	Season	23_A1	US-7 like	13_A2	36_A2	43_A1
2007	Autumn	8	5	0	0	0
2008	Spring	0	4	0	0	0
2008	Autumn	2	5	0	0	0
2009	Spring	1	11	0	0	0
2009	Autumn	9	2	0	0	0
2010	Spring	8	9	7	0	0
2010	Autumn	12	0	0	0	0
2011	Spring	11	3	3	0	0
2011	Autumn	56	0	0	0	0
2012	Spring	5	0	1	0	0
2012	Autumn	10	0	0	0	0
2013	Spring	10	0	1	0	0
2013	Autumn	5	0	0	0	0
2014	Spring	20	2	0	0	0
2014	Autumn	29	0	0	0	0
2015	Spring	26	6	0	0	0
2015	Autumn	26	12	1	0	0
2016	Spring	14	1	36	0	0
2016	Autumn	6	0	7	0	0
2017	Spring	63	0	21	0	0
2017	Autumn	41	0	0	0	0
2018	Spring	45	0	0	4	0
2018	Autumn	60	0	0	0	0
2019	Spring	18	0	0	3	0
2019	Autumn	21	0	0	9	0
2020	Spring	46	0	0	44	0
2020	Autumn	46	0	0	1	0
2021	Spring	61	0	0	21	0
2021	Autumn	46	0	1	0	0
2022	Spring	29	0	0	2	0
2022	Autumn	35	0	0	0	0
2023	Spring	4	0	21	0	0
2023	Autumn	45	0	0	0	0
2024	Spring	25	0	16	0	1

## Data Availability

The data that support the findings of this study are available from the corresponding author upon reasonable request.

## References

[1] Cohen Y., Reuveni M. (1983). Occurrence of metalaxyl-resistant isolates of *Phytophthora infestans* in potato fields in Israel. Phytopathology.

[2] Cohen Y., Farkash S., Reshit Z., Baider A. (1997). Oospore production of *Phytophthora infestans* in potato and tomato leaves. Phytopathology.

[3] Cohen Y., Farkash S., Baider A., Shaw D.S. (2000). Sprinkling irrigation enhances production of oospores of *Phytophthora infestans* in field-grown crops of potato. Phytopathology.

[4] Dietrich C., Hermann D., McKenzie Y., Cohen Y., Gisi U., Stevenson K.L., McGrath M.T., Wyenandt C.A. (2019). Phenylamides: Market Trends and Resistance Situation 35 Years After First Product Introduction. Fungicide Resistance in North America.

[5] Cohen Y. (2002). Populations of *Phytophthora infestans* in Israel underwent three major genetic changes during 1983–2000. Phytopathology.

[6] Kadish D., Cohen Y. (1989). Population dynamics of metalaxyl-sensitive and metalaxyl-resistant isolates of *Phytophthora infestans* in untreated potato crops. Plant Pathol..

[7] Kadish D., Cohen Y. (1992). Overseasoning of metalaxyl-sensitive and metalaxyl-resistant isolates of *Phytophthora infestans* in potato tubers. Phytopathology.

[8] Gisi U., Cohen Y. (1996). Resistance to phenylamide fungicides: A case study with *Phytophthora infestans* involving mating type and race structure. Annu. Rev. Phytopathol..

[9] Cohen Y., Weitman M. (2023). Mobility of oxathiapiprolin in and between tomato plants. Pest. Manag. Sci..

[10] Cohen Y., Rubin A.E., Galperin M., Ploch S., Runge F., Thines M. (2014). Seed Transmission of Pseudoperonospora cubensis. PLoS ONE.

[11] Li Y., Cooke D.E., Jacobsen E., van der Lee T. (2013). Efficient multiplex simple sequence repeat genotyping of the oomycete plant pathogen *Phytophthora infestans*. J. Microbiol. Methods.

[12] El-Ganainy S.M., Ismail A.M., Soliman M.S., Ahmed Y., Sattar M.N., Chellappan B.V., Cooke D.E.L. (2023). Population dynamics of *Phytophthora infestans* in Egypt reveals clonal dominance of 23_A1 and displacement of 13_A2 clonal lineage. J. Fungi.

[13] Patarroyo C., Lucca F., Dupas S., Restrepo S. (2024). Reconstructing the global migration history of *Phytophthora infestans* towards Colombia. Phytopathology.

[14] Chowdappa P., Nirmal Kumar B.J., Madhura S., Mohan Kumar S.P., Myers K.L., Fry W.E., Cooke D.E.L. (2015). Severe outbreaks of late blight on potato and tomato in South India caused by recent changes in the *Phytophthora infestans* population. Plant Pathol..

[15] Dey T., Dwivedi S.K., Datta S., Cooke D.E.L., Roy S.G. (2024). Understanding the temporal dynamics of invasive late blight populations in India for improved management practices. Phytopathology.

[16] McLeod A., De Villiers D., Sullivan L., Coertze S., Cooke D.E.L. (2023). First report of *Phytophthora infestans* lineage EU23 causing potato and tomato late blight in South Africa. Plant Dis.

[17] Cooke D.E.L., Cano L.M., Raffaele S., Bain R.A., Cooke L.R., Etherington G.J., Deahl K.L., Farrer R.A., Gilroy E.M., Goss E.M. (2012). Genome Analyses of an Aggressive and Invasive Lineage of the Irish Potato Famine Pathogen. PLoS Pathog..

[18] Carboxylic Acid Amides (CAA) Working Group Annual Meeting Season 2022 on January 17th, 2023 Protocol of the discussions and recommendations of the CAA Working Group of the Fungicide Resistance Action Committee (FRAC). https://www.frac.info/docs/default-source/working-groups/caa-fungicides/caa-wg/minutes-of-the-2023-caa-meeting-recommendations-for-2023.pdf.

[19] Minutes of the FRAC OSBPI Working Group Meeting 7 February 2024, Corteva lab, Eschbach, Germany. Participants -Corteva Jean-Luc Genet (Chair) Przemek Szubstarski Mamadou Mboup Syngenta Stefano Torriani David Ranner Irina Metaeva Bayer Jürgen Derpmann Andreas Mehl Christian Zupanc. https://www.frac.info/docs/default-source/working-groups/osbpi-wg/minutes-of-the-2024-osbpi-wg-meeting-recommendations-for-2024---7-jan-24.pdf.

[20] Cortesi P., Ottaviani M.-P., Milgroom M.G. (2004). Spatial and genetic analysis of a flag shoot subpopulation of *Erysiphe necator* in Italy. Phytopathology.

[21] Johnson D.A. (2010). Transmission of *Phytophthora infestans* from infected potato seed tubers to emerged shoots. Plant Dis..

[22] Johnson D.A., Cummings T.F. (2009). Latent infection of potato seed tubers by *Phytophthora infestans* during long-term cold storage. Plant Dis..

[23] El-Ganainy S.M., Iqbal Z., Awad H.M., Sattar M.N., Tohamy A.M., Abbas A.O., Squires J., Cooke D.E. (2022). Genotypic and phenotypic structure of the population of *Phytophthora infestans* in Egypt revealed the presence of European genotypes. J. Fungi.

[24] Kaur A., Doyle D., Cooke D.E.L., Mullins E., Kildea S. (2024). First report of the *Phytophthora infestans* EU_43_A1 clonal lineage and associated PiCesA3 mutation G1105S in Ireland. New Dis. Rep..

[25] Abuley I.K., Lynott J.S., Hansen J.G., Cooke D.E.L., Lees A.K. (2023). The EU43 genotype of *Phytophthora infestans* displays resistance to mandipropamid. Plant Pathol..

